# Pollen allergic disease: pollens and its major allergens

**DOI:** 10.1016/S1808-8694(15)31005-3

**Published:** 2015-10-19

**Authors:** Ernesto Akio Taketomi, Mônica Camargo Sopelete, Priscila Ferreira de Sousa Moreira, Francisco de Assis Machado Vieira

**Affiliations:** a PhD, Post-Doctorate. Full Professor; b PhD, FAPEMIG Scholarship; c MS student; d Specialist, Full Professor. Federal University of Uberlândia

**Keywords:** italian rye grass, pollinosis, pollen

## Abstract

Patients with grass pollen allergy, commonly called pollinosis, often present reactivity to pollen allergens from a number of grass species due to cross-reactivity of IgE antibodies to pollen proteins present in pollen grasses. In this context, Italian rye grass (Lolium multiflorum) pollen of the Poaceae family cultivated in Southern Brazil has been considered a major sensitizing agent in patients with pollinosis. In this region, Italian rye grass is capable of producing a great amount of pollen. In addition to L. multiflorum, other Poaceae grasses are naturally grown as weed in Southern Brazil, but with no clinical relevance. Pollen extracts derived from homologous or heterologous grasses are often used for both diagnosis and treatment of seasonal allergy. However, no standardized L. multiflorum pollen extract is commercially available in Brazil and mixed grass extracts are commonly used for diagnosis and immunotherapy of grass pollen allergy. Further studies are required to better characterize the cross-reactivity between L. multiflorum and other grass pollen allergens for improving the diagnosis and immunotherapy to L. multiflorum pollen allergy.

## INTRODUCTION

Pollinosis, also known as seasonal allergic rhinitis, pollen allergy or hay fever, is the result of sensitization to pollen components. The pollen allergens produce clinical symptoms after contact with the airway mucosa and the conjunctiva of previously sensitized individuals. The expression “hay fever” is a misnomer, as in most cases there is neither hay nor fever.

Plants of the Poaceae family are the main source of grass pollen allergens, due to their worldwide distribution and their significant pollen-producing capability^1^. The most important species are Lolium perenne (rye-grass), Poa pratensis (Kentucky bluegrass) and Phleum pratense (timothy grass or herdgrass), but other species may also be clinically significant, depending on the geography^2^.

In Brazil, Lolium multiflorum, also known as Italian rye-grass, is the main cause of pollinosis. However, other allergenic grass species grow haphazardly in city suburbs and on abandoned plots of land, such as Anthoxanthum odoratum (sweet vernal grass), Cynodon dactylon (bermuda grass), Holcus lanatus (common velvet grass), Paspalum notatum (bahia grass) and Bromus sp, among others^3^.

Developments in studies on allergen sensitization and characterization have increased our understanding of the grass pollen allergen sensitization process in sensitive individuals.

The following review details the main features of pollinosis, its diagnosis and prophylaxis, and the main grass pollen allergens related to this condition.

## A REVIEW OF LITERATURE

Charles Blackley is credited with confirming the association between pollen and pollinosis; in 1873 he introduced the skin and provocation tests that confirmed the disease etiology^4^. The first paper on pollinosis in Brazil was published in 1908, where the author, doctor A. Carini from Sao Paulo, questioned the existence of hay fever in Brazil^5^. Further studies have shown a significantly higher incidence of pollinosis caused by grass pollen allergens in atopic individuals in Southern Brazil. However, the extension of grass pollen allergen pollinosis in Brazil is not well known, particularly regarding L. multiflorum, or rye-grass, as in past decades pollinosis was considered rare or non-existent.

In one of the first Brazilian epidemiological surveys, Lima et al. detected skin sensitivity to grass pollen in 0.5% of 2,890 airway allergy cases, while Mendes et al. (1958) observed moderate reactions to pollen in 10.4% of 186 allergy patients in Sao Paulo. The latter also concluded that pollinosis existed in Brazil, and tended to be non-apparent, masked by other forms of sensitization that altered its typical clinical picture^5^.

In a recently published paper, Vieira et al., using the International Study of Asthma and Allergies in Childhood (ISAAC) questionnaire, modified and validated for the city of Curitiba, estimated the prevalence of pollinosis in university students as 22.1% in the city of Caxias do Sul, and 14.1% in the city of Santo Angelo^6^.

Thus, many factors may have been responsible for the development and increased incidence of pollinosis in Brazil, including the introduction of grasses with highly allergenic pollen, deforestation, land use and population increases in areas with well-defined climatic seasons^3^.

### Pollinosis

Clinically, pollinosis is characterized by rhinoconjunctivitis and/or bronchial asthma. Patients present ocular pruritus with conjunctival hyperemia, coryza, sneezing, nasal or pharyngeal-palatal pruritus, and nasal obstruction or the lack thereof. Bronchial hyper-reactivity with associated asthma may be present in 15% to 20% of patients. Conjunctival hyperemia and ocular pruritus and almost always present in pollinosis, which differentiates this condition from the common cold. Airway examination reveals an inflammatory reaction including nasal mucosa edema, augmented turbinates and a transparent mucosal secretion. The nasal secretion cytogram may show an increased number of eosinophils, usually above 10%^5^.

An important feature of pollinosis is annual periodicity, with symptoms usually occurring at the same time of the year, during pollination^3^. In Southern Brazil, symptoms usually start in September and worsen during October and November, in some cases extending into December and January^5^. Some pollen-sensitized patients report allergy symptoms before and after the pollen-releasing season, due mostly to allergens present in households.

Pollen allergen sensitization may occur in isolation or associated with sensitization to other perennial allergens, such as household dust mite allergens (genus Dermatophagoides), fungi, and animal and cockroach epithelium. Thus, symptoms may occur only during spring, the pollen season, or throughout the year (in this case symptoms are worse during spring)^5^. Pollen allergy may be non-apparent and masked by other sensitization processes, which may alter the clinical picture in certain cases^5^.

Repetition of the classical symptoms of rhinoconjunctivitis associated or not with bronchial asthma in two or more pollen seasons strongly suggests pollinosis. The use of a mixed extract containing pollen from different grass species has been recommended, as there may be cross-reactivity between grasses^7^. Nasal or bronchial provocation tests with pollen antigens and specific IgE dosages are ancillary diagnostic methods^5^.

Crude pollen extracts are frequently used for the diagnosis (skin tests) and for specific immunotherapy with allergens, although in the case of grass pollen, allergen potency may vary according to the environmental plant cultivation conditions in species of the same sub-family, 2 the degree of maturity of pollen grains, the extraction procedure and the extract stability^8^.

Prophylaxis is extremely difficult in pollinosis. It is difficult to reduce or avoid environmental exposure, as people work and play in that same environment. A further issue is the maintenance of allergens in households after the pollen season. When the quantity and propagation of pollen in the atmosphere is significant, such as in dry, warm and windy days, patients are advised to remain in closed environments, if possible with filtered air conditioning, and to use glasses when riding bicycles or motorbikes. Other actions include keeping car windows closed, avoiding walks in country clubs, and grass-cutting or gardening^5^.

### Pollen and its allergens

The pollen grain is part of the flowering plant life cycle, and is a specialized structure that harbors the flowering plant male gametes. Its biological function is to fertilize the female gametophyte^4^. Pollen in nature has a variety of shapes (mostly variations of a sphere) and sizes (12 to 300mm diameter). The external wall (the exine) is composed of sporopollenin. The exine is important for the physical and chemical strength of pollen, and covers the pollen grain except along the germ aperture, where it is absent or vestigial. Different from the exine, the inner wall or intine is smooth and does not provide structural support to shape the pollen grain. The intine surrounds the pollen cytoplasm, which contains the intracellular organelles including the vegetative and germ nuclei, starch grains and reduced polysaccharide particles^4^.

In a dry atmosphere pollen may remain stable for centuries. Anemophilous pollen (in which wind-mediated pollination takes place) has allergenic importance. In general, a pollen grain may be transported for 175 kilometers at a velocity of 10 meters/second and will sediment in still air at an approximate average velocity of 3.1 cm/second^9^.

Pollen allergens are water-soluble proteins or glycoproteins, which make them readily available biologically, being capable of evoking an IgE antibody-mediated allergic reaction in seconds. Allergenic particles are expelled from the cytoplasm by at least two suggested mechanisms. In the first mechanism, allergens rapidly diffuse when the pollen grain is in direct contact with the mucosa in an isotonic medium, leading to immediate allergic symptoms on the accessible mucosa surfaces such as the conjunctiva and the nose. In the second mechanism a hypotonic medium (such as rain water) allows rapid hydration of the pollen grain which expels allergen-containing inhalable materials that, due to their reduced size, reach lower airways and induce asthma^10^. Thus, allergen release from pollen grains is a prerequisite for its effect in sensitized individuals.

Pollen grain allergen release may also occur in two separate compartments: on the surface of the upper airway mucosa following pollen exposure, and in ambient air, external to the organism. Therefore, different from house dust mite allergens, the pollen allergen sensitization risk may not adequately be estimated based on a count of external environmental levels.

There are at least three environmental factors that induce pollen allergen release in the air: a high relative humidity, heavy rain and pollutants. In high air humidity, allergens are released from the pollen grain in a process similar to that which occurs in physiological pollinating conditions. Rarely, such as in thunderstorms, pollen grains may rupture as a result of osmotic shock, releasing allergen-containing particles. This finding, as reported in Australia^10^, ^11^, may explain the high frequency of asthma crises during heavy rainfall, which has been suggested as a risk factor for asthma crises^12^.

Grass pollen grains have diameters between 20 to 55mm, and are unlikely to reach lower airways to cause allergy. Grass pollen allergens have been found in association with smaller particles. These particles are small enough to reach the lower airways and therefore may cause allergic reactions in the distal portions of the lung.

Currently, environmental pollutants, especially diesel engine exhaust particles, have been considered as significant pollen allergen releasing factors in the air. These particles contain minerals such as silica, iron, aluminum, magnesium, manganese, sulphur, and others. According to Knox et al. (1997), pollen allergens associated with carbon particles from diesel engine fumes (DECP) would concentrate many allergic molecules in a single particle, as described with the L. perenne Lol p 1 allergen^13^.

Pollen grains release allergens in conditions other than high humidity or hydration. Behrendt et al. (2001) showed that pollen grains may secrete significant amounts of eicosanoid-like substances (substances that cross-react with leukotriene B4 and prostaglandin E2) depending on the pH, time and temperature. The pollen grain, therefore, could itself activate the airway mucosa epithelium by the secretion of pro-inflammatory mediators^14^.

When hydrated, pollen grains may also release a variety of enzymes, including proteases. These proteases are biologically important as they may cause epithelial damage, and are not inactivated by endogenous protease inhibitors^15^. Protease release may generally cause rupture of epithelial junctions, facilitating protein transport, which in turn may sensitize individuals, resulting in increased allergen access to antigen-presenting subepithelial dendritic cells^16^.

### Grass species producing allergenic pollen

Various conditions are required for a plant to cause pollinosis. It must be anemophilous, that is, capable of distributing pollen by the wind, it should have allergenic pollen in sufficient quantities, and it should be close to man^17^.

Thus, a variety of pollen-producing grasses have been recognized as allergenic, including Lolium perenne, Poa pratensis, Phleum pratense, Dactylis glomerata and Cynodon dactylon^7^. Lolium perenne and related grasses are significant sources of allergens in temperate climate regions in North America, Europe and in parts of Australia^1^, ^18^, ^19^.

Worldwide, at least 40% of allergic patients are sensitized to grass pollen allergens^1^, ^20^. In Brazil grass pollen is responsible for almost all cases of pollen allergy; tree and herb pollen is less important in sensitizing atopic individuals and inducing pollinosis in this country. Tree species in Southern Brazil, such as Platanus sp, Ligustrum sp, Araucaria, Acacia sp, and Eucaliptus, may produce pollinosis in highly atopic individuals. Ligustrum sp, although not anemophilous, may spread highly allergenic pollen in its immediate vicinity^3^.

Grass pollen allergens may present shared epitopes. Cross-reactivity and structural homology allow the classification of allergens into groups according to the International Union of Immunological Societies - Allergen Nomenclature Subcommittee (IUIS)^21^. Thirteen pollen allergen groups have been described to date. Clinically, group 1 allergens are the most important, and are recognized by approximately 95% of grass pollen sensitive patients, followed by group 5 allergens, which are recognized by up to 85% of these patients^7^. Other clinically relevant allergens are those of groups 2, 3, 4 and 13, which are recognized by over 50% of grass pollen allergic individuals^22^.

Lol p 1 and Lol p 5, the main cloned and sequenced groups 1 and 5 L. perenne allergens^23^, ^24^, ^25^, are located in different compartments: Lol p 1 is found in the cytosol^26^, ^27^ and Lol p 5 is associated with grass pollen starch grains^25^.

Additionally, the occurrence of many antigen components with a similar molecular mass within a same species may be due to isoforms, such as those seen in Lol p 1 and Lol p 5 (isoforms 4 and 8, respectively)^28^. In grasses, isoforms of an allergic protein are equally recognized by IgE antibodies in allergic patients, frequently having the same molecular mass but with a different isoelectric point^24^, ^25^ resulting from post-translation and transcription changes such as glycosylation, hydroxylation, and the presence of cysteine residues.

Although there is cross-reactivity between grass pollen allergens, single allergens from specific species may also occur. This is the case of P. notatum pollen allergens that have limited cross-reactivity with L. perenne and other clinically relevant grass pollen allergens^2^.

The allergen content of crude extracts may vary not only among different grass species and allergen isoforms, but also according to pollen maturity, allergen extraction procedures and extract stability^8^. Quantification of the main allergens contained in extracts and the characterization of the main grass allergens, including isoforms, are important to develop allergen extracts to optimize the diagnosis and immunotherapy in sensitized patients.

### Lolium multiflorum

L. multiflorum, commonly known as rye-grass, belongs to the Poaceae grass family, and is highly allergenic^17^. It is an exotic (non-native) species in Brazil, brought by European immigrants from the Mediterranean region to be used in agriculture. Ecologically, it grows freely in abandoned rural and urban plots of land, in parks and along roadways^17^. It is highly adaptable to local environmental conditions (soil, climate and topography) and has a high nutritional value. Furthermore, it has established itself due to the ease of natural resowing, disease resistance, good seed producing potential and versatility when used in consortium with other crops^3^.

Adaptability and high nutritional value has facilitated the diffusion of rye-grass in temperate and subtropical regions. It was introduced as forage for winter months in Southern Brazil, and is used in consortium with a variety of legumes in the cold season, and with soybean in summer, adding to the income of farmers during these periods^3^.

Currently rye-grass is used as soil coverage for soybean and corn crops following herbicide desiccation in the so-called “direct plantation” or “direct seeding into standing straw”. Part of the cultivated L. multiflorum is allowed to complete the full vegetative and reproductive cycle to obtain seeds, as these can be readily sold. In such circumstances, it is estimated that all anthers open, releasing pollen into the air to fertilize other flowers and to produce more seeds^6^.

Estimates suggest that a hectare (100 × 100 meters) of a rye-grass crop can produce up to 100 kg of pollen, and that a gram of this pollen can contain around 100 million grains. Sensitized highly atopic patients can present symptoms when exposed to no more than 5 to 10 grains/m^3^ of air^3^.

Being an invasive grass, rye-grass grows uncontrollably in non-agricultural regions, such as along roadways, railways, transmission lines, abandoned land plots in cities and even on sidewalks and roads. So, even densely populated cities may have airborne rye-grass pollen grains during the pollination period^3^.

There is only a single not very extensive published paper on L. multiflorum pollen grain allergens by Schäppi et al. (1999), who reported that the Phl p 5 1D11 monoclonal antibody detected by immunoblotting a group 5 allergen in various grass pollen extracts including L. multiflorum^29^. Our group at the Uberlandia Federal University, in collaboration with Professor Francisco de Assis Machado Vieira from Caxias do Sul, has undertaken further studies to characterize allergens and allergen sensitization to L. multiflorum pollen components. These papers are still to be published, but show that L. multiflorum is adequate to study grass allergen sensitization, particularly due to its importance in causing pollinosis in Brazil.

In our studies L. multiflorum pollen extracts are an useful tool to assess the IgE antibody response against L. multiflorum pollen allergens in pollinosis patients, both in skin puncture tests and in immunoenzyme assays (ELISA) to detect specific serum IgE in pollinosis patients (unpublished results). Furthermore, this extract showed significant cross-reactivity with L. perenne extracts and with commercially available mixed extract grass pollen allergens for skin puncture tests in Brazil.

## DISCUSSION

Many studies have shown an association between grass pollen allergens and atopic disease. Lolium perenne pollen grains have been associated with seasonal allergic rhinoconjunctivitis or pollinosis in temperate climate countries, and its main allergens have been immunocytochemically characterized and located. Other studies have investigated allergen sensitization in L. perenne, an important cause of pollen allergy in temperate climate countries.

Lolium multiflorum is the main pollinosis causing grass in Southern Brazil. L. multiflorum has been used to study grass allergen sensitization in Brazil due to its importance in causing pollinosis-related allergic symptoms in this country. However, its true scope in causing grass pollen allergen pollinosis is still not well known, as most studies are restricted to Southern Brazil. Studies to characterize L. multiflorum allergens are few in Brazilian and world scientific literature. A better definition of grass pollen cross-reactivity would be useful for the specific diagnosis and treatment of pollinosis.

At present there is no standardized (or not standardized) commercial L. multiflorum extract for clinical use in skin test or in specific immunotherapy. Due to cross-reactivity between grass allergens, extracts of grasses not currently cultivated or present in Brazil have been used in the skin puncture test diagnosis of pollinosis. However, further studies are needed to characterize L. multiflorum allergens that show cross-reactivity with other grasses and to assess the relevance of less frequent allergens that could be responsible for pollen allergy in specific allergy patients.

In vitro assays to measure L. multiflorum allergen specific IgE antibodies are not available in clinical laboratories. These could be developed if L. multiflorum pollen extracts were commercially available. Thus, standardized and characterized allergenic extracts need to be developed for specific allergens for medical and laboratory use.

## FINAL COMMENTS

Pollinosis in Brazil has not been given significant importance. However, the development of crude extracts of the main grass pollen allergens related to pollinosis in Brazil should be considered to improve the understanding of pollen disease in this country.
